# Female breast cancer mortality in relation to puberty on Staten Island, New York

**DOI:** 10.3934/publichealth.2020029

**Published:** 2020-06-08

**Authors:** Alfred M Levine, Donna B Gerstle

**Affiliations:** 1 Center for Environmental Science, College of Staten Island, Staten Island, New York, USA; 2 Engineering and Environmental Science, College of Staten Island, Staten Island, New York, USA

**Keywords:** breast cancer, puberty, mortality, case-control, window of susceptibility, obituaries, risk, long latency

## Abstract

Pursuant to a Congressional act in 2008, the Department of Health and Human Services established the Interagency Breast Cancer and Environmental Research Coordinating Committee to address the burden of breast cancer in the United States. Subsequently, the Committee recommended researchers study the timing of exposure to breast cancer risk factors. Given the high breast cancer mortality rate on Staten Island, this paper presents a case-control study investigating breast cancer risk associated with puberty while living on Staten Island. The dataset combined New York City Department of Health and Mental Hygiene female death certificate information between 1985 and 2006, with life history information from newspaper obituaries. Data analyzed included: age, length of residence on Staten Island, birth on Staten Island, and residence on Staten Island during puberty. Cases were individuals who died of breast cancer and controls were individuals who died of non-malignant causes. Analysis included multivariate logistic regression on the full dataset and multiple replicates of randomized one case to two controls simulations. Results indicated that living on Staten Island during puberty (ages 9–19) was associated with an elevated risk of dying from breast cancer (odds ratio 1.35, p < 0.001, 95% CI = 1.18, 1.55). This paper suggests the importance of studying puberty as a window of susceptibility for breast cancer risk.

## 1. Introduction

Staten Island (SI), Richmond County, NY, is one of the five boroughs of New York City (NYC). It is 153 square kilometers in area, located at the southern tip of New York State, approximately 8 kilometers from the borough of Manhattan [1] and has a population of approximately one-half million people [2].

Environmental exposures have been a topic of conversation on Staten Island since 1882 [3]–[7]. SI is downwind from the New Jersey petrochemical industry [8], home to the former Brookfield Landfill [9] and, for more than 50 years, contained the Fresh Kills Landfill, an open dump that received most of NYC's garbage [10],[11]. To address concerns about drinking water quality, an underground storage tank was completed in 1970 [12] and a chlorination facility was opened in 1974 [13].

The New York City Department of Health and Mental Hygiene (DOHMH) has responded to the concerns of the SI community with three studies examining health consequences of environmental exposures [14]–[16]. DOHMH “determined that breast cancer rates on Staten Island have been about 8% to 13% higher than the rest of NYC for many years” [16]. Comparing the five boroughs of NYC, SI had the highest average breast cancer incidence and mortality rates for the period of our study (1985–2006) (See Appendix 1 in the supplementary materials) [17].

While the risk for breast cancer increases with age and the majority are diagnosed after age 50 [18], the most consequential environmental exposures may occur in earlier stages of a woman's life. Indeed, “the breast is especially sensitive to environmental exposures during fetal development (when the organ is formed), and during puberty and pregnancy” [19]. Breast cancer studies have begun to look at these critical stages during a woman's life and how environmental exposures may determine breast cancer outcomes [20]–[32]. In 2019, the International Premenopausal Breast Cancer Collaborative Group published a study analyzing breast cancer risk after recent childbirth [33]. They found that breast cancer risk peaked 5 years after childbirth, before decreasing 34 years after childbirth. The overall pattern was driven by estrogen receptor-positive breast cancer [33].

To address the “burden of breast cancer” in the United States, Congress passed Public Law Section 110–354 (Breast Cancer and Environmental Research Act-BCERA) in 2008 [34]. BCERA established the Interagency Breast Cancer and Environmental Research Coordinating Committee (IBCERCC) to compile past environmental and genomic factors as they relate to breast cancer and make suggestions for future research where there are gaps. The committee suggested researchers “recognize that the timing of exposure to lifestyle and risk factors matters” [19]. In particular, the report “discusses “windows of susceptibility” during the life course when specific exposure(s) might have their greatest influence on lifetime breast cancer risk (e.g., in utero, puberty)” [19].

The Center for Environmental Science at the College of Staten Island/CUNY, an outgrowth of the Master of Science in Environmental Science, has a mission to serve as a university and community focus for scientific, educational, and legal solutions to environmental problems. Inspired by the recommendations of the IBCERCC and responding to the concerns of the SI community and government about the high breast cancer rates on SI, the Center established the Staten Island Breast Cancer Research Initiative (SIBCRI).

Puberty is a complex process with great variability in timing [35]. For the purpose of this paper, the age of puberty was defined as ages 9 through 19 [35]. The aim of this paper was to investigate breast cancer mortality risk associated with exposures during puberty on SI.

## 2. Methods

A retrospective study investigating the risk of SI female breast cancer mortality during the years 1985–2006, using a case-control design, combined life history information from DOHMH death certificates with the *Staten Island Advance* [38] obituaries. Life history information for this paper was defined as an individual's attributes (e.g., sex, date of birth, date of death, cause of death, SI residence history) and was obtained for 923 cases and 10,722 controls.

Cases and controls were determined from death certificates by the NYC Department of Health and Mental Hygiene. Cases were identified as SI females with an International Classification of Disease (ICD) designation of malignant neoplasm of breast, either ICD 9 code 174 [36] or ICD 10 code C50.9 [37]. Controls were SI females who died from causes other than malignant neoplasms. For both cases and controls, the year of birth was 1895 through 1974, and the year of death was 1985 through 2006. Age, defined as the difference between year of death and birth, varied from 25 through 90 inclusive. Cases and controls had to live on SI for at least one year.

Additional life history information was obtained from obituaries in the *Staten Island Advance*, a local newspaper providing information to the SI community since 1886 [38]. A death notice interview was conducted using a pre-established, questionnaire that remained unaltered for decades. The person interviewed, most commonly a surviving spouse or family member, was contacted by a reporter within 24 to 48 hours of an individual's death. The interview was done by an individual trained as a reporter to minimize bias. An analysis of the reliability of obituary data has been published in a previous paper [39].

database. Comparisons were made, and all discrepancies were corrected by referring to the source material. To ensure quality control, all data were double entered by independent staff in a relational.

To examine “windows of susceptibility”, data were analyzed for risk using the following attributes: age, length of residence on SI (SI Years), born on SI (SI Birth = yes/no), and puberty on SI (SI Puberty = yes/no). The odds ratio (OR) for each attribute was defined as the ratio of cases to controls having that attribute divided by the ratio of cases to controls not having that attribute. SPSS (Statistical Package for the Social Sciences, IBM Corporation for Windows) was used for calculating odds ratios from contingency tables. The dataset analyzed is available in Appendix 2 of the supplementary materials.

In this study, age was an important confounding variable [19]. The first approach, using EGRET statistical software for Windows, version 2.0.31, was to include all 923 cases and 10,722 controls in a statistical model in order to find the best fit to a mathematical formula (a logistic curve) that determines the likelihood of an individual dying from breast cancer. This technique (multivariate logistic regression) yields a value for both the odds ratio of cases to controls for each risk factor as well as the 95% confidence interval for the odds ratio.

A second approach to include age as a variable was to use an age-matched case-control design (MedCalc statistical software for Windows, version 17.9.7). The large number of controls in the dataset allowed for a simulation of this technique with multiple random subsets of the entire dataset. A case was selected at random and then matched to two randomly picked age-matched controls. Logistic regression was performed on every random subset. The average of the odds ratios obtained as well as the standard deviation of these ratios was calculated. Details of this technique are available in Appendix 3 of the supplementary materials.

The study received approval from the CUNY Institutional Review Board and underwent review through IRBnet.org.

## 3. Results

### 3.1. Descriptive statistics

Table 1 displays descriptive statistics of the individuals' life history attributes of the study dataset. The average age of cases was 66 whereas the average age for controls was 75. Thirty-five percent of cases and 30% of controls were born on SI. Fifty percent of cases and 39% of controls resided on SI during puberty. The average number of years for length of residence on SI was 45 for cases and 49 for controls.

**Table 1.** Life history attributes for SI female cases and controls 1985–2006.

	Number	Average Age	SI Puberty		SI Birth		Average SI Years
			Number	%	Number	%	
Cases	923	66	463	50	324	35	45
Controls	10722	75	4235	39	3226	30	49
Total	11645	74	4698	40	3550	30	49

To analyze puberty risk, a 2 × 2 contingency table was formed (Table 2). The odds ratio was 1.54 (p < 0.001, 95% CI = 1.35, 1.76). *X^2^* (1, 11645) = 40.2, p < 0.001. This analysis, however, does not include the important age differences between cases and controls.

**Table 2.** 2 × 2 contingency table for SI female cases and controls (1985–2006) for puberty risk.

	Cases	Controls	
SI Puberty	463	4235	4698
Not SI Puberty	460	6487	6947
	923	10722	11645

### 3.2. Multivariate analysis

Using multivariate logistic regression on the entire dataset (first approach) with age and SI Puberty as variables, an odds ratio of 1.35 (p < 0.001, 95% CI = 1.18, 1.55) was obtained.

Table 3 displays the results obtained from fifteen random replicates of age-matched cases and controls (second approach). In all replicates every case was used and successfully matched to two random controls. Within each replicate, no control was used more than once. The odds ratios for the 15 random replicates varied from 1.33 to 1.50, with a mean value of 1.43. The standard deviation of the odds ratio was 0.041. Assuming a normal distribution, this corresponded to a 95% confidence interval of 1.35 to 1.51.

**Table 3.** Odds ratios and confidence intervals for 15 random matches (one case to two controls) for SI puberty for SI female cases and controls 1985–2006.

	Confidence Interval		
	Odds ratio	Lower	Upper
Match 1	1.33	1.14	1.56
Match 2	1.39	1.18	1.63
Match 3	1.43	1.22	1.67
Match 4	1.46	1.25	1.71
Match 5	1.42	1.21	1.67
Match 6	1.44	1.22	1.68
Match 7	1.44	1.22	1.68
Match 8	1.45	1.24	1.70
Match 9	1.45	1.24	1.70
Match 10	1.47	1.25	1.72
Match 11	1.40	1.19	1.64
Match 12	1.42	1.21	1.66
Match 13	1.50	1.28	1.76
Match 14	1.46	1.25	1.71
Match 15	1.39	1.19	1.63
Mean	1.43		
SD	0.041		

### 3.3. Confounding variables

The dataset included SI Years and SI Birth as well as SI Puberty. Since there may have been correlations between these variables, multivariate logistic regression was done to investigate whether SI Puberty was the major risk.

Using multivariate logistic analysis with age and SI Years, the odds ratio for length of residence on SI was 1.004 (p < 0.009, 95% CI = 1.001, 1.008). When age, SI Years and SI Puberty were included, the odds ratio for SI Years dropped to 0.997 (p < 0.20, 95% CI = 0.991, 1.002) while the odds ratio for SI Puberty increased to 1.52 (p < 0.001, 95% CI = 1.21, 1.92).

Similarly, using multivariate analysis with age and SI Birth the odds ratio for SI Birth was 1.10 (p < 0.17, 95% CI = 0.95, 1.57). However, when age, SI Puberty, and SI Birth were included the odds ratio for SI Puberty increased to 1.7 (p < 0.001, 95% CI = 1.39, 2.08) while the odds ratio for SI Birth decreased to 0.73 (p < 0.003, 95% CI = 0.59, 0.90).

## 4. Discussion

This study has investigated the high rate of breast cancer mortality on SI using the variables age, length of residence on SI, born on SI, and residing on SI during puberty years. Since these attributes may not have been independent, the interactions between the variables had to be considered. The main result was that women who went through puberty on Staten Island are 35% more likely to die of breast cancer than women who went through puberty elsewhere.

Because the average age of breast cancer cases were nine years younger than controls, it was apparent that age was needed as a variable in the investigation of risk factors. Since the percentages for cases compared to controls were higher for both SI Puberty and SI Birth an analysis was needed to separate these factors in determining risk. In contrast, since the average length of residence on SI of cases was less than controls, the number of years residing on SI was unlikely to be an important breast cancer mortality risk.

Multivariate logistic regression (first approach), with age and SI Puberty as variables, determined an odds ratio for SI Puberty of 1.35. Adding either length of residence on Staten Island or birth on Staten Island did not improve the accuracy of this result. The analysis established that the 95% confidence interval for the odds ratio was 1.18 to 1.55.

Using the alternate technique (second approach) of simulating random age-matched case-control studies yielded an average odds ratio of 1.43 with a 95% confidence interval of 1.35 to 1.51 (Table 3). The odds ratio obtained was statistically equivalent to the previous result but had a narrower confidence interval. Further mathematical analysis is needed to establish the validity of the second approach, and this will be the subject of a future paper. The two independent techniques concluded that residing on Staten Island during puberty was a significant risk factor.

It is true that 70% of the cases that resided on SI during their puberty years were also born on SI. The multivariate analysis including age, SI Puberty, and SI Birth returned an odds ratio of less than one (negative risk) for SI Birth implying that the important risk was SI Puberty. Furthermore, the dataset included 65 women born on Staten Island who moved away before puberty and then returned to Staten Island. None of these women died of breast cancer. While additional study is needed, these two results suggest that the important exposure was during puberty and not in utero.

Additionally, it is necessary to study Staten Island's unique environmental risks relating to breast cancer to identify possible exposures women had during the window of susceptibility. An unusual geological feature of SI are serpentine outcroppings. This ridge of serpentine makes up approximately one-quarter of the land area [40]. Serpentine outcrops are associated with known carcinogens including asbestos, chromium, and cadmium [41],[42]. Between 1917 and 1970, SI drinking water was stored in the Silver Lake reservoir which sat on top of serpentine rock. Starting in 1970, the drinking water was stored in reinforced concrete tanks holding 100 million gallons [12],[40].

The Fresh Kills Landfill was established by the City of New York on SI [43] in 1948 and became the largest open dump [11] in the world. For over 50 years, until its closure in 2001, this landfill received most of New York City's household solid waste [43],[44]. Bordering Fresh Kills Landfill was Brookfield Landfill (a designated Superfund site), where it was determined that illegal toxic waste dumping occurred [45],[46]. Contaminants to human health included inorganics (metals), volatile organic compounds (VOCs), semi-volatile organic compounds (SVOCs), pesticides and polychlorinated biphenyls (PCBs). Recently DOHMH concluded that a “... proximity analysis did not suggest that breast cancer rates were higher near the landfill….” [16].

The authors have concluded that there are several limitations to this study. Death certificate data was obtained from DOHMH in November 2008. Due to the complex nature of the dataset, merging various attributes required unanticipated extra resources and time. This study covered breast cancer mortality between the years 1985 and 2006. The results should be confirmed with studies on more recent time periods. Additionally, there are limitations associated with using obituary data. The two main reasons for a person who died on SI with a DOHMH death certificate but no *SI Advance* death notice was the family's desire for privacy or funeral arrangements off Staten Island. This was true for both cases and controls. The result in this paper should be corroborated by other studies where residence during puberty can be established by an alternate approach.

## 5. Conclusions

This paper examined breast cancer mortality risk associated with residing on Staten Island during puberty. Two independent statistical approaches obtained equivalent results suggesting that puberty was a significant window of susceptibility. This conclusion supports the recommendation of IBCERCC that researchers study the timing of exposure to breast cancer risk factors [19]. The current analysis strongly suggests that the mechanism occurred during puberty and not in utero. Additionally, this study indicated that the location of the individual during puberty affected breast cancer mortality approximately half a century later. This confirms the conjecture that there is a long latency period between the exposure and the outcome of breast cancer [23].

While this study has identified a window of susceptibility for breast cancer risk for Staten Island, it has not identified specific environmental exposures associated with that risk. It is possible that the improvements in environmental quality over time have reduced the breast cancer mortality risk faced by current Staten Island females during puberty. The next step in studying this question is to identify other geographic areas with similar environmental perturbations that have high breast cancer incidence and mortality rates to determine if there is a commonality with Staten Island.
