# Supplementary material for: Female breast cancer mortality in relation to puberty on Staten Island, New York
Source: AIMS Public Health. 2020 Jun 8;7(2):344–53. doi: 10.3934/publichealth.2020029 (PMC7327401; doi:10.3934/publichealth.2020029)
Supplement: Supplementary file 3 [file publichealth-07-02-029-appendix3.pdf]

Public Health

AIMS Public Health, 7(2): 344–353.
DOI: 10.3934/publichealth.2020029
Received: 19 May 2020
Accepted: 04 June 2020
Published: 08 June 2020

http://www.aimspress.com/journal/aimsph

Research article

Female breast cancer mortality in relation to puberty on Staten Island, New York

Alfred M Levine[1,2] and Donna B Gerstle[1,*]

[1] Center for Environmental Science, College of Staten Island, Staten Island, New York, USA
[2] Engineering and Environmental Science, College of Staten Island, Staten Island, New York, USA

* **Correspondence:** Email: Donna.Gerstle@csi.cuny.edu; Tel: +718-982-3922.

Appendix 3. Staten island breast cancer research initiative double match program procedure

To analyze the confounding variable of age, one technique used was an age-matched case-control study where each case was matched with two randomly chosen control individuals with the same age.

To obtain the first match, cases and controls were assigned a random number generated by the random number function in Microsoft Excel (RANDBETWEEN). A random case was selected. The case-control matching procedure in MedCalc for Windows, version 17.9.7 was used to match this case to a random control with the same age. The selected control was removed from the dataset and the procedure was repeated for the remaining cases.

To obtain a second match, controls that were not used during this first match and all cases were copied back into Microsoft Excel. These cases and controls were again assigned a random number generated by the random number function in Microsoft Excel. These individuals were put back in MedCalc and the second match was made.

This set of 2–1 matches constituted a single replicate. We calculated 15 random replicates and reported median results as well as maximum and minimum odds ratios.

AIMS Press

2020 the Author(s), licensee AIMS Press. This is an open access article distributed under the terms of the Creative Commons Attribution License (http://creativecommons.org/licenses/by/4.0)
